# The Causality Research between Syndrome Elements by Attribute Topology

**DOI:** 10.1155/2018/9707581

**Published:** 2018-07-02

**Authors:** Tao Zhang, Mengqi Liu, Wenyuan Liu

**Affiliations:** ^1^School of Information Science and Engineering, Yanshan University, No. 438, Hebei Avenue, 066000 Qinhuangdao, China; ^2^Hebei Key Laboratory of Information Transmission and Signal Processing, No. 438, Hebei Avenue, 066000 Qinhuangdao, China

## Abstract

**Background:**

The traditional Chinese medicine (TCM) is an empirical medical system and has its own diagnosis and treatment method. The syndrome elements are atoms to modern TCM diagnosis proposed by Professor Zhu Wenfeng. Researching and analyzing the syndrome element system is one of the active issues for TCM research. At present, most related researches focus on the correlativity and hierarchical relationship of the diseases and symptoms, but the causality researches between syndrome elements themselves have not been reported so far.

**Methods:**

To explore the causality between syndrome elements, a method named causality by attribute topology (CAT) is proposed. Based on the subordinate relations in attribute topology, the inference method analyzes and reasons the dependency relationship between the sets of objects which contain attributes. Through the removal of attributes in the attribute topology, the formal context is updated constantly. Thus, the causal relationship among the attributes is deduced. In this method, 500 records are mathematically transferred to a binary context for syndrome element analysis. Through the analysis and verification of the potential causal relationship between the syndrome elements, knowledge discovery of the diagnostic data of traditional Chinese medicine based on attribute topology structure diagram is conducted.

**Results:**

This paper has verified the causal transformation between these syndrome elements. The experimental results between the female group data and the male group data show that different genders have different characteristics and relations of syndrome elements. The experimental results are basically consistent with the traditional Chinese medicine theory.

**Conclusion:**

The experiment shows that causality by attribute topology (CAT) is feasible to describe the causality between TCM syndrome elements. Further research on possible knowledge discovery in TCM diagnostic data should be conducted through the analysis of the potential causal relationship between TCM diagnostic data and each syndrome element.

## 1. Introduction

In the field of traditional Chinese medicine (TCM), treatment based on syndrome differentiation is the basis for preventing and treating diseases [1, 2]. As the premise of TCM treatment, the accuracy of syndrome differentiation will have a necessary influence on the effect of treatment [3, 4]. The fundamental inference methods of traditional Chinese diagnostics include the following: to infer inner changes from outer phenomenon, to deduce overall status from partial changes, and to identify syndromes in the standard of a healthy person [5]. These are classic methods for the discovery of syndrome elements in TCM, which have been fully studied by Zhu [6] and applied to clinical diagnosis by Hong [7, 8].

Zhu [9, 10] introduced the term “syndrome element” based on the research of syndrome differentiation and quantitative correlation relation between pairs of syndrome elements in TCM. “Syndrome element” was defined [11] as the basic element of syndrome differentiation; the identification of “syndrome” to determine disease location and disease-natures; the basic element of “syndrome name.” Following the previous studies, he further studied the syndrome elements of disease-natures and disease location and proposed a novel system of syndrome differentiation based on syndrome elements [12]. This new system identifies syndrome elements from clinical symptoms and then determines the syndrome name according to the identified syndrome elements [13]. Therefore, the relationship between syndromes, syndrome elements, and the syndrome name has become the focus of the study on the syndrome element system [14].

Zhu [6, 15, 16] obtained the standard weights between syndromes and syndrome elements by double frequency weight scissors fork algorithm. Li Candong [17–22] discussed the correlation between five viscera identification and facial lesion distribution. Five viscera have a relative position in the face. The disease location of puberal acne is closely related to liver and kidney. Xiong Liping [23] analyzed a lot of cases and found that syndrome elements have an influence on syndromes. Dai [24] found and verified that there is a relationship between pale tongue and some syndrome elements. Hong analyzed the relationship between syndromes, syndrome elements, and syndrome names by the principle of attribute partial order and formed a syndrome analysis system which further standardized the syndrome differentiation system. According to the theory of traditional Chinese medicine, there is a certain causal relationship between syndrome elements, such as the syndrome element Yin Deficiency and the syndrome element Exterior which are both the causes of the syndrome element Fire-Heat. However, the mathematical analysis of the causal relationship between syndrome elements has not been reported yet.

As a branch of attribute partial order, attribute topology [25–28] is a tool focused on formal concept analysis [29, 30], cognitive computing, and relationship analysis [31–34]. In this paper, we propose a causal inference method by attribute topology under the representation framework of attribute partial order graphs. The method is applied to clinical data analysis, and the causal relationship between syndrome elements in clinical data is derived, which will be the basis for further knowledge discovery in syndrome elements system [35–38].

## 2. Materials and Methods

### 2.1. Attribute Topology

Attribute topology (AT) and attribute partial ordering graph belong to the framework of formal structure analysis, which is a graph description for formal context. Formal context, which acts as the research object and data representation, is an important basic aspect in FCA. Here are a few notions about formal context.

**Definition 1 .**

A formal context *K* = (*G*, *M*, *I*) consists of two sets *G* and *M* and a relation *I* between *G* and *M*. The elements of *G* are called the objects and the elements of *M* are called the attributes of the context. In order to express that an object *g* is in a relation *I* with an attribute *m*, we write *g*Im or (*g*, *m*) ∈ *I* and read it as “the object *g* has the attribute *m*”.

**Definition 2 .**

In a formal context *K* = (*G*, *M*, *I*), for *A*⊆*G* of objects, *f*(*A*) = {*m* ∈ *M*∣∀ *g* ∈ *A*, (*g*, *m*) ∈ *I*}. Correspondingly, for *B*⊆*M* of attributes, *g*(*B*) = {*g* ∈ *G*∣∀ *m* ∈ *B*, (*g*, *m*) ∈ *I*}.

From the perspective of graph theory, attribute topology shows a weighted graph that depicts the relationships between attribute pairs. Thus the storage method of the graph can be borrowed. This section carries out a description of adjacency matrix of AT from the perspective of inclusive relationship of attribute pairs.

**Definition 3 .**

In context *K* = (*G*, *M*, *I*), *AT* : = (*V*, *Edge*) is defined as adjacency matrix of AT in which *V* = *M* is the set of vertex in AT and Edge represents the weight of edges in AT. Edge is expressed as follows:

(1) Edgemi,mj=⌀,gmi∩gmj=⌀⌀,gmi∩gmj=gmigmi∩gmj,others

### 2.2. Attribute Topology and Causal Analysis

By the definition of attribute topology, the attribute topology itself emphasizes the correlation between attributes. At the same time, the relationship between superordinate attributes (SPA) and subordinate attributes (SBA) provide a way for causal analysis.

**Definition 4 .**

In context *K* = (*G*, *M*, *I*), *m*_*i*_ ∈ *M* and *m*_*j*_ ∈ *M*, *i* ≠ *j*. *m*_*i*_ is the subordinate attribute of *m*_*j*_ and satisfied *g*(*m*_*i*_) ⊂ *g*(*m*_*j*_).

From the definition of SBA, Property 5 is included obviously.

**Property 5 .**

In context *K* = (*G*, *M*, *I*), *m*_*i*_ ∈ *M*, *m*_*j*_ ∈ *M*, and *m*_*i*_ is the SBA of *m*_*j*_; then *m*_*i*_ is a necessary condition for *m*_*j*_.

**Definition 6 .**

In context *K* = (*G*, *M*, *I*), *m*_*i*_ ∈ *M*, *m*_*j*_ ∈ *M*, and *m*_*i*_ is a necessary condition for *m*_*j*_; then *m*_*j*_ is part cause of *m*_*i*_ and *m*_*i*_ is the result of *m*_*j*_, recorded as *m*_*j*_ → *m*_*i*_.

**Definition 7 .**

In context *K* = (*G*, *M*, *I*), *m*_*m*_, *m*_*n*_, *m*_*p*_, *m*_*q*_, *m*_*i*_ ∈ *M* and *m*_*m*_ → *m*_*i*_, *m*_*n*_ → *m*_*i*_, *m*_*p*_ → *m*_*i*_, *m*_*q*_ → *m*_*i*_. There is no *m*_*k*_, which makes *m*_*k*_ → *m*_*i*_ and then set *m*_*c*_ = {*m*_*m*_, *m*_*n*_, *m*_*p*_, *m*_*q*_} is cause of *m*_*i*_, the subsets of *m*_*c*_ is the part cause of *m*_*i*_, and *m*_*i*_ is the result of *m*_*c*_, recorded as (*m*_*m*_, *m*_*n*_, *m*_*p*_, *m*_*q*_) → *m*_*i*_.

**Property 8 .**

In context *K* = (*G*, *M*, *I*), *m*_*i*_ ∈ *M*, *m*_*j*_ ∈ *M*, and *m*_*j*_ → *m*_*i*_; then *g*(*m*_*i*_) − *g*(*m*_*j*_) = Φ and *g*(*m*_*j*_) − *g*(*m*_*i*_) ≠ Φ.

**Proof**

∵*m*_*j*_ → *m*_*i*_,* ∴g*(*m*_*i*_)⊆*g*(*m*_*j*_),* ∴g*(*m*_*i*_) − *g*(*m*_*j*_) = Φ, and *g*(*m*_*j*_) − *g*(*m*_*i*_) ≠ Φ.

**Definition 9 .**

In context *K* = (*G*, *M*, *I*), the vertex whose outdegree is 0 and the nonzero indegree is the leaf node and its attribute is called leaf attribute.

**Property 10 .**

In context *K* = (*G*, *M*, *I*), *m*_*i*_ ∈ *M* and *m*_*i*_ is a leaf attribute, and its set of causes is the set of all adjacent vertices in the attribute topology.

**Proof**

According to Definition 9, in context *K* = (*G*, *M*, *I*), *m*_*i*_ ∈ *M* and *m*_*j*_ ∈ *M*. *m*_*i*_ is a leaf attribute and *m*_*j*_ is the adjacent vertex of *m*_*i*_. From Definition 4 and Definition 6, the conclusion *m*_*j*_ → *m*_*i*_ is obtained obviously. So the set of causes is *m*_*i*_.

### 2.3. The Algorithm

According to the theory of the previous section, the algorithm of causal analysis by AT is designed as follows.

**Step 1 .**

Getting *AT* : = (*V*, *Edge*) by a context, if there are leaf nodes, proceed to Step 2; otherwise, proceed to Step 4;

**Step 2 .**

If there is *m*_*j*_ → *m*_k_, the set of causes *m*_*c*_ is calculated for *m*_*k*_. *m*_*c*_ is a set of attributes that are not null in the set of matrices *Edge*(*m*_*i*_, *m*_*j*_). Then get causality *m*_*c*_ → *m*_*i*_.

**Step 3 .**

Update the context, *K* = (*G*, *M*, *I*), *G* = *G* − *g*(*m*_*i*_), *M* = *M* − *f*(*m*_*i*_), and *I* = *G* × *M*, and then go to Step 1, until there is no relationship *I* = *G* × *M*.

**Step 4 .**

Finish.

Here is an example.

**Figure 1:**
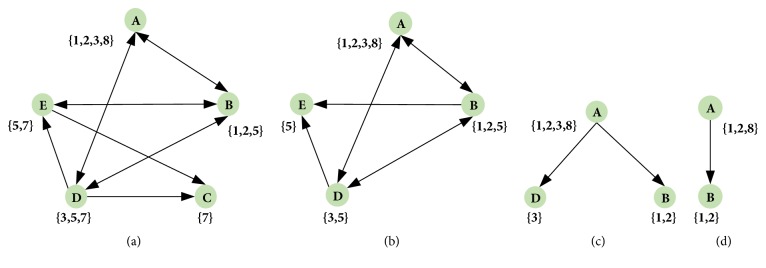
The attribute topology. (a) The AT of Table 1, (b) the AT of Table 2, (c) the AT of Table 3, and (d) the AT of Table 4.

For a context as Table 1, its AT is Figure 1.

In Figure 1(a), the attribute *C* is a leaf attribute obviously, and the cause of *C* is {*D*, *E*}. As a result of that, there is (*D*, *E*) → *C*. Update the AT by Step 3, *G* = *G* − *g*(*C*) = {1,2, 3,4, 5,6, 7,8}−{7}. Then the updated context is Table 2 and its AT is Figure 1(b).

In Figure 1(b), the attribute *E* is a leaf node and can conclude (*B*, *D*) → *E* by Property 10. Update Table 2, the context shown in Table 3, and the AT shown in Figure 1(c).

In Figure 1(c), the attribute *D* is a leaf and the cause of *D* is {*A*, *B*}. Then get the following causality: (*A*) → *D*. Update Table 3, and then get the context shown in Table 4 and the AT shown in Figure 1(d).

In Figure 1(d), the attribute *B* is a leaf and the cause of *B* is {*A*}. Then get the following causality: (*A*) → *B*. And the causality between all attributes in the attribute topology can be inferred; there is (*D*, *E*) → *C*, (*B*, *D*) → *E*, (*A*) → *D*, and (*A*) → *B*.

### 2.4. Methods

#### 2.4.1. Date Acquisition

The experimental data in this paper are from the clinical data, collected by the team of Hong [39]. In the system, for the clinical manifestations mapping to syndrome elements, 177 inquiry questions (related to symptoms or signs common in clinical diagnosis) are designed for males, while 194 inquiry questions are designed for females. 670 medical records were analyzed by Hong team. According to the method of Section 2.4.2 Data Processing, the data is filtered and 500 valid data are retained as the experimental data of this paper. A total of 500 cases are collected, of which are 300 males and 200 females and their age distribution is 40 to 70 years old. The syndrome element date we have measured in the experiment includes forty-five indicators: Yang Deficiency, Yang Hyperactivity, Exterior, Qi Deficiency, Qi Sinking, Qi Counterflow, Blood Stasis, Blood Cold, Stirring Wind, Retained Fluid, Fluid Depletion, External Wind, Summer Heat, Dryness, Food Accumulation, Gallbladder, Large Intestine, Stomach, Bladder, Small Intestine, Chest and Diaphragm, Sinew and Bone, and so on. The analytical methods involved in the experiment include analysis of eight principle syndrome differentiations [40], analysis of Qi-blood-fluid-humor syndrome differentiation [41], analysis of disease cause syndrome differentiation [42], and analysis of visceral syndrome differentiation [43] in the traditional Chinese medicine.

The data used in this experiment are collected according to the user's online examination in the diagnostic system. Due to some nonstandard behaviors in data storage, the data are incomplete, inconsistent, and noisy. In order to obtain high quality datasets to improve the accuracy of the algorithm, the first task is to preprocess the collected data.

Each row in the experimental data represents questionnaire results by a user. Each column is the measurements for all users under a certain syndrome element.

It is not convenient to display all the original data because the amount of data is large and involves a large number of syndrome elements. The 10 most used syndrome elements are shown in Table 5.

**Figure 2:**
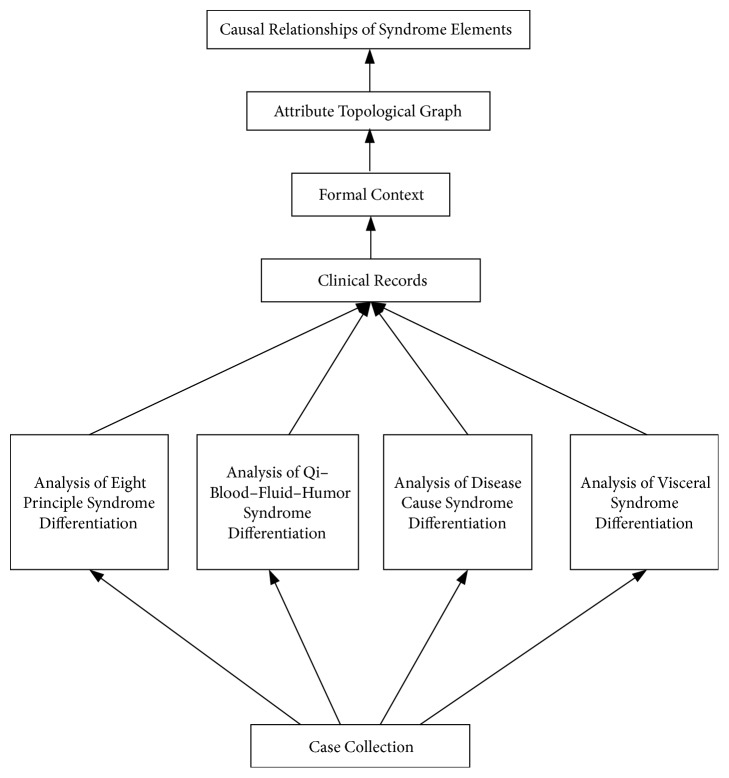
The overall analysis process.

A total of 500 cases are collected and analyzed in four aspects. The collected data are expressed in the form of formal context. The causal relationships of syndrome elements can be obtained through the corresponding attribute topological graph.  Figure 2 shows the overall analysis process.

#### 2.4.2. Data Processing

The data processing method is as follows: (1) if all questions are answered identically by a user through online examination in the diagnostic system (namely all the values of the syndrome elements are the same), only one of these records is taken as valid data for the same answer in this paper (the same data is an invalid data for causal analysis), as shown in No.54 user and No.98 user in Table 6; (2) if some results of measurement are too extreme (i.e., values of all syndrome elements are either maximum or minimum), these results are excluded in data processing as certain patients have made extreme answers, as shown in No.17 user in Table 6; (3) if there are numerous 0 values in the diagnostic result of a user (possibly caused by mistakes in data collection), this record is invalid and should be excluded (it is less possible to obtain such a result through diagnostic system for syndrome elements are interrelated), as shown in No.113 user in Table 6.

As shown in No.53 in Table 6, the value of syndrome element Half-Exterior Half-Interior is 0. Considering that it is possible that the users do not have the relevant feature of this syndrome element, rather than the error caused by data extraction, the above situation has not been removed directly.

Since there is no normal value or normal range of the indicators, it is difficult to discover the above data only according to the size of the data rather than compare with the normal value. In order to reasonably indicate the normal values of the indicators, the method used in this paper is as follows: the average value of all data in each column is used as a critical value of the indicators, greater than or equal to this value has the index considered not normal, and less than this value is considered normal indicators. In order to analyze the impact of gender on the indicators more comprehensively and rationally in this paper, the above raw data is divided into two parts, one part male data and the other female data.

Because the data processed by causal inference method based on attribute topology and qualitative reasoning are formal context, the collected data is expressed in the form of the formal context. The index value corresponding to the attribute of all the date which is greater than or equal to the average value is assigned to 1, and the index value corresponding to the attribute which is smaller than the average value is assigned a value of 0. No.2, No.3, No.5, No.6, and No.7 in Table 7 show the data of female after processing. No.1, No.4, No.9, No.12, and No.13 in Table 7 show the data of male after processing. After the purification (remove duplicate data, remove the row with the same content, etc.) of objects and attributes by formal context, the number of male data changed to 300 sets and the female data changed to 200 sets.

#### 2.4.3. The Cause Analysis

The number of syndromes in the experimental data is relatively large; consequently, the attribute topological graph may be very complex if all the experimental data are converted into attribute topological graph. Taking it into account, this paper chooses the first eight syndrome elements of the first six objects in the inference process as a demonstration.

The original formal context after preconditioning is shown in Table 8.

It can be seen clearly from Table 8 that the values of each syndrome element in object 3 are 0, so object 3 is removed. Syndrome element Cold has the same content as syndrome element Yang Deficiency, so these two can be combined. The formal context after purification is shown in Table 9.

**Figure 3:**
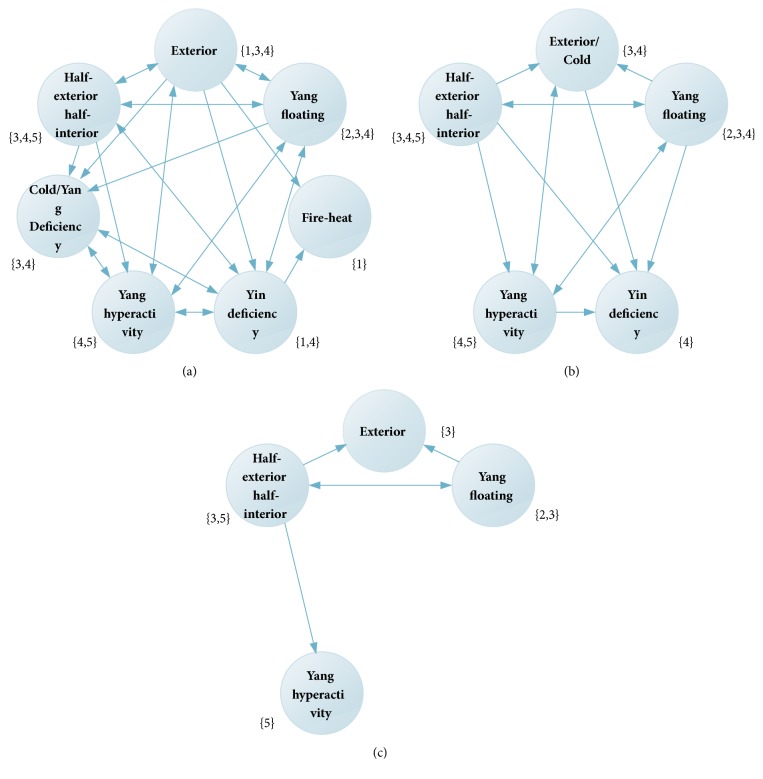
Attribute topological graph. (a) The original attribute topological graph by the conversion of Table 9, (b) the first updated attribute topological graph, and (c) the second updated attribute topological graph.

The formal context shown in Table 9 is transformed into the original attribute topological graph shown in Figure 3(a).

By analyzing the original attribute topological graph, the syndrome element Heat should be the starting syndrome element to infer its causal relationship with other syndrome elements. It can be concluded that the syndrome element Yin Deficiency and the syndrome element Exterior are the cause of the syndrome element Fire-Heat and the syndrome element Fire-Heat is the result.

After the first update, syndrome element Cold and syndrome element Exterior belong to the same set of objects {3,4}, so this paper combines syndrome element Exterior and syndrome element Cold together. The resulting attribute topological graph is shown in Figure 3(b).

After the first update, the causal relationship is inferred: (Exterior, Yin Deficiency)→Fire-Heat. Remove the syndrome elements Fire-Heat, and update the object set of syndrome element Exterior and syndrome element Yin Deficiency. Secondly, the syndrome element Yin Deficiency was selected to judge the causal relationship between itself and other syndrome elements. The attribute topology is updated a second time, and the update results are shown in Figure 3(c). After updating twice, the causal relationship is inferred: (Exterior, Half-Exterior Half-Interior, Yang Hyperactivity, and Yang Floating) →Yin Deficiency. Execute the above update loop until all causal relationships are inferred.

## 3. Results and Discussion

Representation and determination of causality, the relationship between an event (the cause) and a second event (the effect), where the second event is understood as a consequence of the first, are a challenging problem [44]. From the experimental data study, the causality between the syndrome elements in female group is shown in Table 10. The causality between the syndrome elements in male group is shown in Table 11. For example, as shown in the third line of Table 11, Qi counterflow and insecurity of Qi are the causes of syndrome elements. Qi deficiency is the effect of syndrome elements. Generally speaking, Qi counterflow and insecurity of Qi are special characterization of Qi deficiency [45]. This association analysis of syndrome elements can show which syndrome elements appear more frequently and identify possible relationships between syndrome elements.

For ease of analysis, the female group data were expressed as the first set of data and the male data were represented as second set of data. It is found that the experimental results of the two groups of data were extremely different, not only in the relationship between the two syndrome elements, but also in the relationship between the syndrome elements of combination.  Table 12 lists the comparison of the two sets of data.

Due to the two groups of data involved in the number of syndrome elements which is relatively large, Table 12 does not list all the syndrome elements involved.  Table 12 lists relatively few syndrome elements that are not involved, as well as the ratio of its total syndrome elements. It turns out, a total of 12 syndrome elements are not involved in the first group, accounting for 26.67% of the total syndrome elements. There are 12 syndrome elements in the second group which are not involved, accounting for 8.89% of the total syndrome elements. Obviously, there are little syndrome elements which are not involved in the second group compared with the first group; the proportion of the total syndrome element is 8.89%. The syndrome elements which are not involved in the first group account for a high proportion of total syndrome elements, but only 26.67%.

In order to compare two groups of data more intuitively, the inference group number is taken as an index for comparative analysis, shown in Table 13.

The group of inference is the group of syndrome elements with causal relationship inferred from the causal relationship. Analysis shows that the two groups of data inference group are similar; the first group is 26 and the second group is 29. The proportion of syndrome elements involved in the two sets of data is also more than 70%.

These unequal results indicate that the male patient would be more likely to involve more syndrome elements. From these two groups, we can see that the contribution for syndrome elements research of male group was greater than that of female group. In the meantime, it can be inferred that the difference of data between male group and female group is the life habit, skin constitution, viscera function, hormone content, and so on. This kind of analysis would provide an objective basis for standardization of dialectic diagnosis.

Through the above analysis, we can see that the results of causal inference involve fairly comprehensive syndrome elements, which can be used as a data support for further causal analysis. Because of the more combinations of syndrome elements and the lack of theoretical basis for some combinations of syndrome elements, this paper mainly analyzes the relationship among various syndrome elements in the existing mature theory of traditional Chinese medicine: the relationship between the five internal organs. Five internal organs include heart, lung, spleen, liver, and kidney. The relationship between the five internal organs and their relationship in TCM is shown in Table 14.

Tables 15 and 16 give the inference results of two sets of data to analyze the relationship among five internal organs.

It can be seen from Tables 14–16 that although the experimental results are different from the traditional Chinese medicine theory, the whole is basically consistent.

The main cause for the difference is that all the syndrome elements are considered as a whole when proving the causality between each syndrome elements in this paper; however, TCM theory is only analyzed from the five internal organs. There is also a need to combine more clinical data as well as syndromes and syndrome elements for the causal relationship between other syndrome elements.

## 4. Conclusions

In this paper, a visual inference method of causal relation between TCM syndrome elements is proposed based on the theory of attribute topology. The main purpose of this algorithm is to verify the causal transformation of TCM syndrome elements through clinical data collection. Through this experiment, we have preliminarily verified the causal transformation between these syndrome elements. This paper is discussed from the male-female perspective. As for future work, the analysis can further be expanded to account for other aspects of causality.

The next steps include the following: (1) to scale up clinical data collection and extend the mathematical expression of the relationship between TCM syndrome elements and promote the objectivity of TCM; (2) to discover the relationship between syndrome elements uninvolved in the classical literature from the clinical data for the development of the TCM syndrome element system.

## Figures and Tables

**Table 1:** The original formal context.

	A	B	C	D	E
1	X	X			
2	X	X			
3	X			X	
4					
5		X		X	X
6					
7			X	X	X
8	X				

**Table 2:** The first updated formal context.

	A	B	D	E
1	X	X		
2	X	X		
3	X		X	
5		X	X	X
8	X			

**Table 3:** The second updated formal context.

	A	B	D
1	X	X	
2	X	X	
3	X		X
8	X		

**Table 4:** The third updated context.

	A	B
1	X	X
2	X	X
8	X	

**Table 5:** The 10 most used syndrome elements.

Gender	Half-exterior half-interior	Cold	Fire-heat	Yin deficiency	Yang deficiency	Yang floating	Dampness	Phlegm	External wind

**Table 6:** Data analysis.

No.	Gender	Exterior	Half-exterior Half-interior	Cold	Fire-Heat	Yin Deficiency	Yang Deficiency	Yang Hyperactivity	Yang Floating
17	Female	1345.77	5585.71	0.11	0.09	634.36	5647.99	345.91	0.05
53	Male	16.35	0.00	35.06	100.86	123.87	141.78	56.02	19.08
54	Female	10.76	15.28	30.56	42.02	94.69	75.03	32.92	0.00
98	Female	10.76	15.28	30.56	42.02	94.69	75.03	32.92	0.00
119	Female	45.76	0.00	36.21	0.00	0.00	0.00	44.91	0.00

**Table 7:** Part of the female-male data after purification.

No	Exterior	Half-exterior Half-interior	Cold	Fire-Heat	Yin Deficiency	Yang Deficiency	Yang Hyperactivity	Yang Floating
2	0	0	1	1	0	0	0	0
3	0	0	0	0	0	0	0	1
5	0	0	0	0	0	0	0	0
6	1	1	0	0	1	0	1	0
7	1	1	0	1	1	1	1	1
1	1	1	1	1	1	1	1	1
4	0	0	0	0	0	0	0	1
9	1	1	1	1	1	1	1	1
12	0	0	0	0	0	1	1	0
13	1	0	1	1	0	1	0	0

**Table 8:** Original formal context.

No.	Exterior	Half-exterior Half-interior	Cold	Fire-Heat	Yin Deficiency	Yang Deficiency	Yang Hyperactivity	Yang Floating
1	1	0	0	1	1	0	0	0
2	0	0	0	0	0	0	0	1
3	0	0	0	0	0	0	0	0
4	1	1	1	0	0	1	0	1
5	1	1	1	0	1	1	1	1
6	0	1	0	0	0	0	1	0

**Table 9:** The formal context after purification.

No.	Exterior	Half-exterior Half-interior	Cold / Yang Deficiency	Fire-Heat	Yin Deficiency	Yang Hyperactivity	Yang Floating
1	1	0	0	1	1	0	0
2	0	0	0	0	0	0	1
3	1	1	1	0	0	0	1
4	1	1	1	0	1	1	1
5	0	1	0	0	0	1	0

**Table 10:** The causality between the syndrome elements in female group.

**Cause of syndrome elements**	**effect of syndrome elements**
**Heart spirit**	Qi deficiency
**Kidney, Fluid depletion**	Heart
**Blood deficiency, Uterus**	Liver
**Summer heat, Heart, Heart spirit, Retained fluid**	Spleen
**Lung, Fluid depletion, Food accumulation**	Heart
**Exterior**	Yin deficiency
**Fluid depletion, Large intestine**	Fire-heat
**Half-exterior half-interior**	Essence deficiency
**Yang floating**	Qi sinking
**Retained fluid, Small intestine, Bladder**	Yang hyperactivity
**Food accumulation, Blood cold, Heart spirit, Small intestine, Uterus**	Stirring wind
**Summer heat**	Dryness
**Skin**	Fluid depletion
**Water retention, Blood cold, Stirring blood, Uterus**	Blood deficiency
**Food accumulation**	Small intestine
**Summer heat**	Half-exterior half-interior
**Blood cold, Food accumulation, Retained fluid, Half-exterior half-interior**	Lung
**Blood heat, Stirring blood, Uterus**	Blood cold
**Water retention, Uterus**	Stirring blood
**Uterus**	Summer heat
**Blood heat, Stirring blood, Uterus**	Blood cold
**Water retention, Uterus**	Stirring blood
**Uterus**	Food accumulation
**Uterus**	Water retention
**External wind, Retained fluid, Lung, Skin**	Exterior
**Blood heat, Retained fluid, Uterus, Spleen**	Lung
**Yang floating, Blood heat, Uterus, Skin**	External wind
**Uterus**	Skin
**Uterus**	Blood heat

**Table 11:** The causality between the syndrome elements in male group.

**Cause of syndrome elements**	**Effect of syndrome elements**
**Fire-heat, Dryness**	Liver
**Summer heat, Stirring blood**	Blood deficiency
**Qi counterflow, Insecurity of Qi**	Qi deficiency
**Half-exterior half-interior, Yang floating, Dampness, Summer heat, Qi sinking, Water retention, Heart spirit, Heart, Small intestine**	Spleen
**Food accumulation**	Dampness
**Phlegm, Dryness, Fluid depletion**	External wind
**Exterior, Lung**	Liver
**Summer heat, Blood heat**	Yin deficiency
**Summer heat, Qi counterflow**	Fire-heat
**Yang deficiency, Dryness, Essence deficiency, Qi sinking, Insecurity of Qi, Fluid depletion, Heart spirit, Small intestine, Large intestine, Bladder, Skin**	Yang hyperactivity
**Half-exterior half-interior, Uterus**	Heart spirit
**Yang deficiency, Yang floating, Dryness, Qi sinking, Insecurity of Qi, Retained fluid, Lung, Kidney, Bladder, Skin**	Essence deficiency
**Dryness, Fluid depletion, Bladder, Skin**	Qi sinking
**Yang floating, Gallbladder, Uterus**	Yang deficiency
**Skin**	Fluid depletion
**Qi counterflow, Insecurity of Qi, Lung**	Dryness
**Stirring wind, Insecurity of Qi, Lung**	Kidney
**Yang floating**	Gallbladder
**Bladder**	Half-exterior half-interior
**Food accumulation, Uterus**	Small intestine
**Cold, Blood cold, Uterus, Spleen**	Lung
**Meridian–collateral, Sinew and bone**	Cold
**Retained fluid**	Qi counterflow
**Insecurity of Qi, Bladder**	Sinew and bone
**Yang floating**	Bladder
**Stirring wind**	Yang floating
**Insecurity of Qi**	Stirring wind
**Blood cold, Meridian–collateral**	Insecurity of Qi
**Chest and diaphragm**	Uterus

**Table 12:** The comparison of the two group of date sets.

**Group number**	**Syndrome elements not involved**	**Ratio**
**First group**	Cold, Dampness, Qi stagnation, Blood stasis, Insecurity of Qi, Stirring wind, Kidney, Gallbladder, Stomach, Sinew and bone, Chest and diaphragm, Meridian–collateral	26.67%
**Second group**	Qi stagnation, Blood stasis, Stirring blood, Stomach	8.89%

**Table 13:** The comparative analysis after adding inference group number.

**Group name**	**Inference group number**	**Number of syndrome element**	**Ratio**
**First group**	26	33	73.33%
**Second group**	29	41	91.11%

**Table 14:** Five internal organs and their relationship.

**The five internal organs**	**Relationship**
**Heart and Lung**	The heart governs the blood and the lung governs Qi. Although the normal operation of blood is the heart leading, it must promote with the help of the Lung Qi.

**Heart and Spleen**	The heart governs the blood and the spleen governs the blood. The spleen can govern the blood if the function of the spleen is normal.

**Heart and Kidney**	The heart and kidney are interactive and inter-conditioned so as to maintain the relative balance of physiological functions

**Liver and Spleen**	The liver storing blood. The spleen controlling digestion as well as essence of water and grain to produce blood

**Liver and Lung**	The meridian and vessels of liver passes through the fat and is injected into the lung.

**Liver and Kidney**	The liver stores blood, and the kidney stores the essence. Liver blood needs to depend on the nourishment of kidney essence and the kidney essence needs the supplement of liver blood continuously, both are interdependent and mutual promotion.

**Spleen and Kidney**	Spleen Yang rely on warm nourishing of kidney yang to play the role of transportation and transformation.

**Lung and Kidney**	lung governing purification and descending and regulation of water passages so that water metabolism inferior to the kidney

**Lung and Spleen**	lung being reservoir of phlegm. Spleen being source of phlegm.

**Table 15:** The inference results of five internal organs in female group.

**Cause of syndrome elements**	**Effect of syndrome elements**
**Kidney, Fluid depletion**	Heart
**Summer heat, Heart, Heart spirit, Retained fluid**	Spleen
**Lung, Fluid depletion, Food accumulation**	Heart
**Blood heat, Retained fluid, Uterus, Spleen**	Lung

**Table 16:** The inference results of five internal organs in male group.

**Cause of syndrome elements**	**Effect of syndrome elements**
**Half-exterior half-interior, Dampness, Summer heat, Qi sinking, Water retention, Heart spirit, Heart, Small intestine**	Spleen
**Exterior, Lung**	Liver
**Stirring wind, Insecurity of Qi, Lung**	Kidney
**Cold, Blood cold, Uterus, Spleen**	Lung

## Data Availability

The data used to support the findings of this study are included within the article.

## Abbreviations

TCM: Traditional Chinese medicine
FCA: Formal concept analysis
AT: Attribute topology.
